# 2701. Mycobacterial Immune Reconstitution Inflammatory Syndrome Following Hematopoietic Cell Transplantation for GATA2 Deficiency

**DOI:** 10.1093/ofid/ofad500.2312

**Published:** 2023-11-27

**Authors:** Brian P Epling, Jennifer Cuellar-Rodriguez, Danielle Arnold, Daniele Avila, Thomas Bauer, Kristen Cole, Janine Daub, Lisa Duncan, Juan Gea-Banacloche, Stella Ma, Roxanne Merkel, Cindy Palmer, Christa Zerbe, Steven M Holland, Irini Sereti, Dennis Hickstein, Maura Manion

**Affiliations:** NIH/NIAID, Washington, District of Columbia; National Institute of Allergy and infectious Diseases, Bethesda, MD; NCI/NIH, Bethesda, Maryland; NCI/NIH, Bethesda, Maryland; NCI/NIH, Bethesda, Maryland; NCI/NIH, Bethesda, Maryland; NCI/NIH, Bethesda, Maryland; NCI/NIH, Bethesda, Maryland; NIAID/NIH, Bethesda, Maryland; NIAID/NIH, Bethesda, Maryland; NCI/NIH, Bethesda, Maryland; NIH/NIAID, Washington, District of Columbia; Laboratory of Clinical Immunology and Microbiology, National Institute of Allergy and Infectious Diseases, National Institutes of Health, Bethesda, MD; National Institutes of Health, Bethesda, Maryland; Laboratory of Immunoregulation, NIAID, Bethesda, MD; NCI/NIH, Bethesda, Maryland; Division of Clinical Research, NIAID, Bethesda, MD

## Abstract

**Background:**

Immune reconstitution inflammatory syndrome (IRIS) was first described in patients with HIV and opportunistic infections starting antiretroviral therapy, but may be seen following hematopoietic cell transplantation (HCT) in patients with pre-existing infections. GATA2 deficiency is a genetic disorder with a marked susceptibility to mycobacterial infections, and is treated definitively with HCT. The clinical factors and pathogenesis of IRIS in HCT require further investigation.

**Methods:**

We conducted a retrospective review of 32 patients with GATA2 deficiency and mycobacterial infections who completed > 6 months of follow-up after HCT. Uniform criteria were used to define cases of IRIS (Image 1). IRIS cases were classified as either early (post-HCT day < 60), intermediate (days 61-120), or late (day > 120).

**Image 1.**
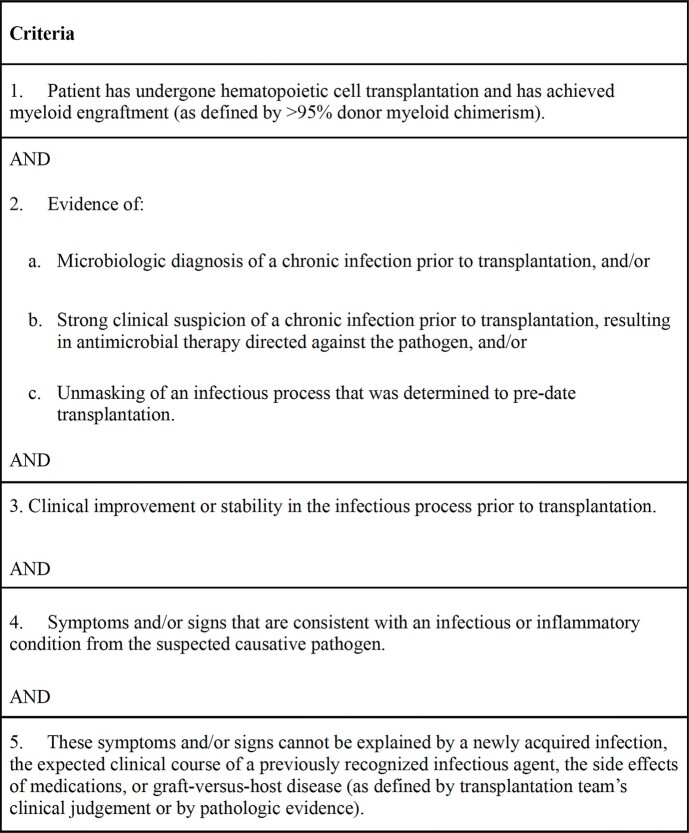
Post-Hematopoietic Cell Transplant IRIS Criteria

Criteria were adapted to hematopoietic cell transplantation from the AIDS Clinical Trial Group IRIS criteria.

**Results:**

Clinical characteristics are shown in Image 2. IRIS was identified in 12 (35%) patients (Images 2-3). These patients were more likely to have disseminated infection (P=0.03), an elevated pre-HCT ferritin (P=0.03), and a lower day 30 post-HCT CD4 count (P=0.04).

Median time to IRIS was 100 days (Image 4). Pre-transplant duration of mycobacterial therapy correlated with time to IRIS (Pearson’s r=0.58, P=0.05). Conditioning regimens using total body irradiation with or without cyclophosphamide were associated with longer time to IRIS (hazard ratio=0.04, P=0.02). A rise in CD3 chimerism was seen during early cases of IRIS, but not intermediate to late (P=0.002). IRIS emerged during tapering of systemic immunosuppressive therapy in 33% of cases. IRIS was managed conservatively (50%), by intensification of anti-mycobacterial treatment (8%) or by intensification of immunosuppression (42%).

**Figure:**
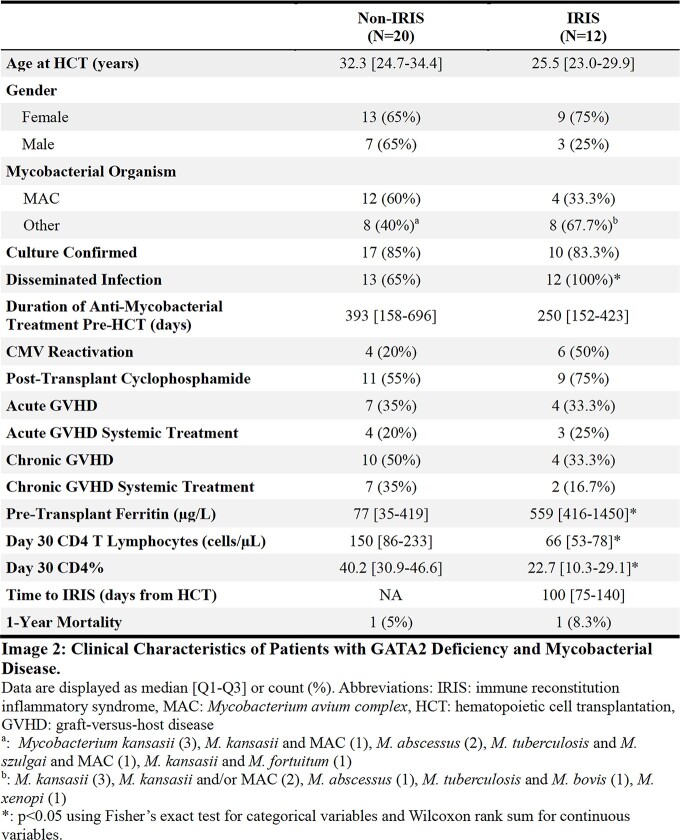

**Figure:**
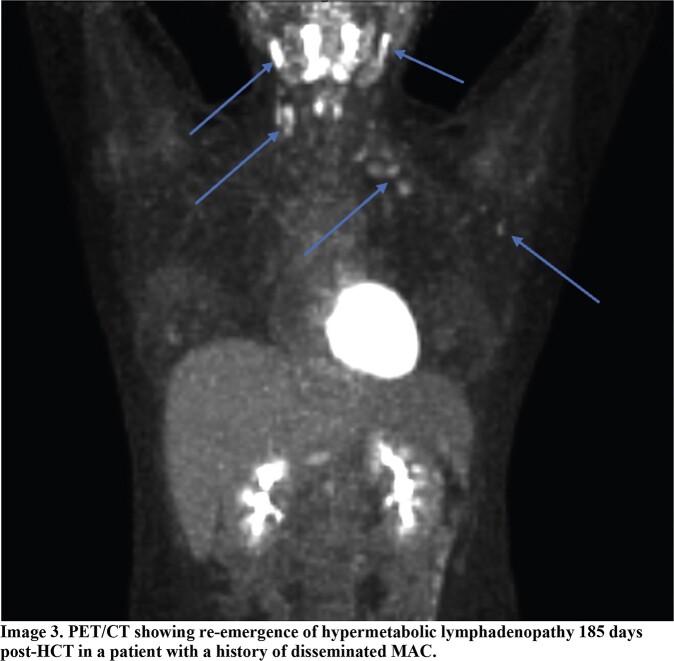

**Figure:**
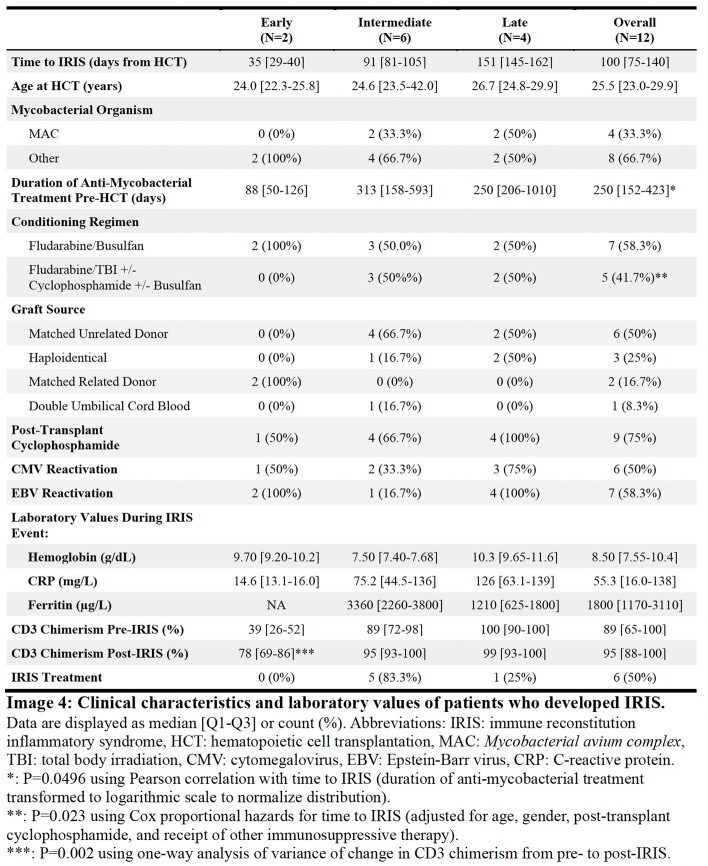

**Conclusion:**

Development of mycobacterial IRIS after HCT for GATA2 deficiency was associated with disseminated infection, a high pre-transplant ferritin, and low day 30 CD4 count. These findings suggest that antigen burden, a hyperinflammatory state leading into transplantation, and CD4 nadir after HCT have a role in its pathogenesis. Early cases of IRIS may emerge during lymphoid engraftment and CD4 recovery, whereas late cases may emerge with decreases in immunosuppressive therapy and more intensive conditioning regimens. Clinical outcomes were similar to non-IRIS patients.

**Disclosures:**

**All Authors**: No reported disclosures

